# Depression in multicultural Australia: Policies, research and services

**DOI:** 10.1186/1743-8462-4-16

**Published:** 2007-07-23

**Authors:** Harry Minas, Steven Klimidis, Renata Kokanovic

**Affiliations:** 1 Centre for International Mental Health, School of Population Health, The University of Melbourne, Bouverie Street, Carlton, Victoria 3053, Australia; 2 The Victorian Transcultural Psychiatry Unit, St. Vincent's Health Melbourne, Nicholson Street, Fitzroy, Victoria 3065, Australia

## Abstract

**Background:**

Depression is one of the leading causes of disability in Australia. The cultural and linguistic diversity of the Australian population poses a significant challenge to health policy development, service provision, professional education, and research. The purpose of this study is to explore the extent to which the fact of cultural and linguistic diversity has influenced the formulation of mental health policy, the conduct of mental health research and the development of mental health services for people with depression from ethnic minority communities.

**Methods:**

The methods used for the different components of the study included surveys and document-based content and thematic analyses.

**Results:**

Policy is comprehensive but its translation into programs is inadequate. Across Australia, there were few specific programs on depression in ethnic minority communities and they are confronted with a variety of implementation difficulties. The scope and scale of research on depression in Ethnic minority communities is extremely limited.

**Conclusion:**

A key problem is that the research that is necessary to provide evidence for policy and service delivery is lacking. If depression in Ethnic minority communities is to be addressed effectively the gaps between policy intentions and policy implementation, and between information needs for policies and practice and the actual research that is being done, have to be narrowed.

## Background

Depression, one of the leading causes of disability worldwide, [1-3] will be the second largest cause of disability in Australia by the year 2020 [3]. Suicide, often related to depression, is the fourth largest cause of mortality in Australia [3]. The Australian Government recognises the need to address the issue of depression [4-7] and several initiatives are under way, the most notable being *beyondblue: the national depression initiative *[8]. The approach of *beyondblue *is to foster sustainable partnerships among organisations, agencies, service providers, community and government sectors, individuals, consumers and caregivers, to promote coordinated activities and to build on existing strengths in order to address the problem of depression in the Australian community. The purpose of *beyondblue *is reduce the burden of depression. However, it is not clear whether these and other activities will deal effectively with depression in a multicultural and multilingual population. An adequate response to depression in this context will rely on three broad components: a body of mental health and services research to guide policy and practice, relevant policies, and resources for services that translate good policies into practice. This study reports findings from a national examination of policy, research and service delivery in relation to depression in ethnic minority communities in Australia.

Australia is home to many ethnic, religious and language groups. Broad indicators of diversity show that 28% of the population was overseas-born (15% in a non-English speaking country) at the time of the 2001 Census and 16% of the population spoke a language other than English at home. Ethnic minority communities vary substantially with respect to mean age, time of migration, language, religion, type of immigration, along many other variables. There is also significant social and economic diversity within any particular ethnic minority community. Diversity poses a significant challenge in the areas of health service policy, service provision, professional education and research.

Despite Australia's adoption of multiculturalism as national policy over the past 35 years, it is only in the last decade that mental health services have attended to the issue of linguistic and cultural diversity. Some important developments are the inclusion, as part of the National Mental Health Strategy, of cultural diversity as an important component of the National Standards for mental health service delivery [9], the establishment of specialist state transcultural mental health centres, specialist services for refugees, and the establishment of a national transcultural mental health network [10]. New models of care are being explored, for example bilingual clinical services [11,12]. Such developments have occurred in the context of the major reform of mental health systems with the closure of large psychiatric hospitals and the development of community-based services and more recently, a concentrated effort to engage general practice in mental health care.

In view of the composition of the Australian population it is important that mental health services adequately respond to the complexity created by the interactions between culture, language and mental health. Cultural factors play a significant role in depression (and other mental disorders), particularly in how the illness is experienced, the personal meaning of the illness, clinical manifestations, how it affects help-seeking and pathways to care, and in issues such as adherence and responsiveness to treatment [13-20]. In multilingual settings, additional concerns relate to the problems of translation and the dynamics of the interpreting process, and the increased probability of diagnostic error and poor treatment outcomes [17,21-24].

Attention to cultural and linguistic diversity is an important consideration in tackling the problem of depression in Australia. The aim of this study was to examine the current status of policy, research and service provision in Australia in relation to depression and ethnic minority communities. We explore the extent to which an appropriate policy framework has been developed, the extent, nature and usefulness of Australian-based research, and the existence of service programs for depression that are responsive to the needs of ethnic minority communities.

We do not here advocate for any particular response to the challenges presented to the mental health system by the cultural and linguistic diversity of the Australian population. (For example, we do not argue for or against ethnospecific mental health services.) Our purpose is to examine the developments in the areas of policy, research and services that have occurred in response to the reality of cultural and linguistic diversity since the beginning of the national mental health strategy.

## Methods

### Policy Analysis

Major Commonwealth and State/Territory mental health policies and related documents were examined for their relevance to mental health system responses to depression in Ethnic minority communities. The policies examined are listed in Table 1. Content analysis (described in the Results) was used in examining them.

**Table 1:** List of Commonwealth/National and State Policies examined

**LOCATION**	**MENTAL HEALTH POLICY TITLE**
Commonwealth	National Mental Health Policy, 1992 [66]
Commonwealth	Mental Health Statement of Rights and Responsibilities, 1991 [67]
Commonwealth	National Standards for Mental Health Services, 1996 [9]
Commonwealth	Second National Mental Health Plan, 1998 [68]
Commonwealth	Mental Health Promotion and Prevention National Action Plan Under the Second National Mental Health Plan: 1998–2003, 1999 [69]
Commonwealth	National Action Plan for Promotion, Prevention and Early Intervention for Mental Health, 2000 [4]
Commonwealth	Youth suicide in Australia: the national youth suicide prevention strategy, 1997 [70]
Commonwealth	National Action Plan for Depression, 2000 [6]
Commonwealth	Life – A framework for prevention of suicide and self-harm in Australia, 2000 [7]
Australian Capital Territory	The future of Mental Health Service in the Australian Capital Territory – Moving Towards 2000 and Beyond – A Whole of Territory Strategic Plan 1998–2001, 1998 [71]
New South Wales	Caring for Mental Health – A Framework for Mental Health Care in NSW, 1998 [72]
	Caring for Older People's Mental Health – A Strategy for the Delivery of Mental Health Care for Older People in NSW, 1998 [73]
	Caring for Mental Health in a Multicultural Society – A Strategy for the Mental Health Care of People from Culturally and Linguistically Diverse Backgrounds, 1999 [28]
	Getting in Early – A Framework for Early Intervention and Prevention in Mental Health for Young People in NSW, 2001 [74]
Tasmania	Tasmanian Multicultural Policy, 2001 [75]
	Rural Mental Health Plan, 2001–2004, 2001 [76]
	A Plan for Now and the Future – Strategic Plan for 1999–2002, 1999 [77]
Western Australia	Making a Commitment – The Mental Health Plan for WA, 1996 [78]
	Transculturally Orientated Mental Health Services, 2001 [26]
Queensland	Queensland Health non-English speaking background mental health policy statement, 1995 [27]
	Ten year mental health strategy of Queensland, 1996 [79]
	Queensland Health Multicultural Policy Statement, 2000 [80]
South Australia	A New Millennium – A new beginning, 2000–2005 – Mental Health in South Australia, 2000 [81]
Victoria	Victoria's Mental Health Services: Framework for service delivery, 1996 [31]
	Improving services for people from a non-English speaking background, 1996 [29]
	New Directions for Victoria's Mental Health Services: The next five years, 2002 [82]

### Research Analysis

#### Publications

Medline and Psychinfo searches were undertaken for publications by Australian researchers, including all publications where any of the authors had an affiliation to an Australian institution. Search terms were combinations of the following: depression and/or suicide and cultural and/or ethnic and/or language and/or immigrant and/or refugee groups. The search was limited to publications in the period 1990 – the starting point for the national mental health strategy – to 2002, when the data collection was concluded. Only original research was included. Papers were summarised for details related to depression or suicide.

#### Higher Degree Theses

Research theses from 1990 onwards were sought. An internet search was conducted of 30 Australian tertiary institution libraries using 'advanced' key word search facilities, where available, for the terms depression or suicide and thesis. We counted relevant theses as those describing in their title a focus on immigration or, ethnic or language minority groups in Australia.

#### Databases

The Social Science and Data Archives (SSDA) [25] held by the Australian National University, provides information regarding Australian databases available for secondary analysis. Databases were screened to identify those with information potentially relevant to depression or suicide and ethnic minority communities. Further information was sought from the websites of the Australian Bureau of Statistics (relating to the Australian Survey of Mental Health and Wellbeing and the National Health Surveys) and the Department of Immigration and Citizenship (DIAC) (formerly the Department of Immigration, Multicultural and Indigenous Affairs), relating to the Longitudinal Surveys of Immigrants to Australia. Sixty-nine databases were admitted to more detailed content analysis of their abstracts and variables.

#### Research Funding

Major Australian funding organisations, including the National Health & Medical Research Council (NH&MRC), Australian Research Council (ARC), VicHealth, HealthWay, and Australian Rotary Health Fund were asked to provide information on their funding priorities and currently funded projects. Websites and annual reports were inspected for relevant information. Documentation was sought for the five years prior to the time of our assessment. All materials were interpreted and relevant content aggregated on priorities and currently funded projects.

#### Current Research Activities

Two hundred and seventy-seven relevant university departments and research organisations were identified through university websites and directories and were surveyed to supply information on relevant research. Heads of organisations/departments were invited to send the questionnaires we supplied to any researchers known to be conducting relevant work. The questionnaire asked about the nature of the research; funding support; collaborations; research team size and disciplines; involvement of ethnic communities, caregivers and consumers; location of the work; current status of the work; and publications.

### Service Programs and Projects

A nationwide survey was conducted in capital cities and major regional towns to identify specific services addressing depression in ethnic minority communities. Questionnaires were sent to 1480 organisations including ethnic community organisations, mental and general health service providers, Divisions of General Practice, public health units, Local Governments, Migrant Resource Centres, transcultural mental health services, refugee and other services. Those identifying a relevant program on a screening questionnaire were invited to provide more detailed information about potentially relevant programs or projects. All survey responses were examined as to whether the programs were specific to mental health, depression and the level of focus on ethnic minority communities. Relevant programs were analysed in relation to reported strategies and activities, barriers, supports, perceived role in depression in ethnic minority communities, partnerships and program involvement of ethnic communities, caregivers and consumers.

## Results

### Policies

Preliminary examination indicated that specialised State 'transcultural mental health' policies [26-29] provided comprehensive policy coverage of issues relevant to mental health and ethnic minority communities. It should be noted that policies were almost invariably concerned with broad issues of mental health and illness and did not focus specifically on depression. Key topics (Table 2) were identified from these policies and used to content analyse each of the major Commonwealth and State/Territory mental health policies. The examination of the policies indicated a highly variable degree of attention to issues relevant to ethnic minority communities.

**Table 2:** List of issues addressed by State Transcultural Mental Health Policies

◦ access and equity
◦ effectiveness and efficiency
◦ coordination, continuity of care
◦ mental health services/service providers
◦ staff development/education/training
◦ planning which meets the community's needs
◦ collaborations and partnerships with ethnic minority communities, consumers and caregivers
◦ delivering culturally sensitive services
◦ community education/support
◦ providing information which supports access
◦ interpreters/language services
◦ general practice
◦ support for ethnic minority community workers
◦ assuring quality
◦ data collection
◦ research
◦ service utilisation
◦ monitoring and evaluation
◦ the role of specialised transcultural mental health services
◦ mental health promotion
◦ special target groups

Against the topics covered by the transcultural mental health policies, Commonwealth policies provided a relatively comprehensive coverage of issues. Areas that were unrepresented or under-represented included: Providing information which supports access; Interpreters/language services; Coordination of care; Support for ethnic community workers; Data collection; and Service utilisation. More recent policies (since 1995) have tended to include a clearer focus on ethnic minority communities and call for improvement of the evidence base for all forms of mental health activity in relation to ethnic minority communities.

At the time of the study there was no single policy at the Commonwealth level dedicated to the mental health of ethnic minority communities. Reference to ethnic minority communities in mainstream policies has been at the most general level, and strategies advocated for the 'special needs groups', including immigrants and refugees, were often separated from the main strategy. Approximately half of the policies examined contain a brief mention of ethnic minority communities, often as an 'after-thought', once the general policy model for the mainstream community has been developed.

All of the State mainstream mental health policies make reference to ethnic minority communities but Victoria, New South Wales, Western Australia and Queensland (referred to as 'active states' below) have sections dedicated to ethnic minority communities. These same States have explicit Transcultural Mental Health policies and have had active involvement with the Australian Transcultural Mental Health Network, now operating as Multicultural Mental Health Australia. They also fund specialist Transcultural Mental Health services that are dedicated to mental health service improvements relating to ethnic minority communities. These policies give clear indications of how services may be modified to respond appropriately and effectively to the needs of ethnic minority communities.

More than the Commonwealth policies, State policies consider the strategies by which improvements in service provision are to be pursued. These strategies are intended to support the work of local area-based mental health services in improving services to ethnic minority communities. Less well covered areas in the State mental health policies in relation to ethnic minority communities are issues of: Effectiveness and efficiency; Planning based on community needs; Community education and support; and Data collection, monitoring and evaluation.

Within the active States the specialist transcultural mental health policies represent a comprehensive statement of the range of required service improvements and directions. Apart from offering clear direction to mental health services, they focus on community engagement, development, and education in relation to mental health, and the inter-linking of mental health services with community-based organisations. The specialist transcultural policies also take advantage of State-based knowledge and resources in developing local service improvements.

Despite the existence of a well-developed policy environment in Australia in relation to mental health and ethnic minority communities, there remain substantial problems in policy implementation [30]. *Victoria's Mental Health Services – The Framework for Service Delivery *[31] identifies this problem as one of motivation for reform: "...the general lack of service response to persons from different cultural backgrounds comes not so much from a lack of knowledge about what should be done but more perhaps from a lack of will to do it....it is long past the time when health providers did not have to ensure that their services are able to be provided in a culturally sensitive manner." (p. 28)

### Research

#### Publications

Research on depression within ethnic minority communities in Australia is predominantly in three areas: research on refugees and asylum seekers (10 studies); studies related to the post-partum period (6 studies); and research on suicide (6 studies). Additional papers dealt with a range of relevant issues, but not specifically on depression in ethnic minority communities (8 studies).

In the period surveyed the most developed program of depression-relevant research was that on refugees [32-37]. This work has explored pre- and post-migration factors associated with psychological morbidity in addition to information on depression, anxiety and post-traumatic disorders. The second consistent area of work has been on post-natal depression [38-43], but limited to Arabic-speaking and Vietnamese-speaking groups. The subject of suicide received some attention [44-48], but this work has been entirely based on suicide rates in various birthplace populations and no work has been done on psychosocial factors in suicide, suicide risk and behaviours.

The remaining eight studies [49-56] cover different issues, including levels of depression, correlates of depression, epidemiology as part of the National Survey of Mental Health and Wellbeing, general practitioner and patient agreement on mental health status, depression in general practice patients, effectiveness of training community workers, depression in women with young children, adolescents, and international students.

#### Higher Degree Theses

Examination of the 228 identified theses on depression in Australia since 1990 revealed only five theses (2.2 percent) addressing issues relevant to ethnic minority communities [57-61].

#### Databases

Examination of accessible databases indicated that few were relevant for the purposes of secondary analyses in relation to ethnic minority communities and depression. More common were databases that included information on immigrants' living conditions in Australia, including social contacts, family structure, employment, housing, perceived prejudice, physical health and a range of migration and settlement factors. These factors could be useful to analyse in as far as they can be considered as determinants (risk and protective factors) or correlates of depression. Most of the data however are old and may be of more theoretical value than providing a picture of contemporary issues confronting immigrants. The Longitudinal Surveys of Immigrants, developed by DIAC, may also give some indication of causal pathways through the possible examination of associations of variables across several follow-up data collections, but these data provide information only for recent arrivals to Australia.

#### Research Funding

The assessment of funding priorities and funded projects revealed that most funding bodies provided some scope within their statements of priority to for grant submissions for mental health research on ethnic minority communities. Currently funded projects included NH&MRC projects examining suicide trends, refugee psychiatric status and service utilization, and the risk factor profile of the New South Wales Vietnam-born community; an ARC grant to study the narratives of migrants in cultural transition; and a HealthWay grant to examine depression and mental health promotion within ethnic minority and indigenous groups. The VicHealth funding round of 2001 was exceptional, supporting eight projects focusing on selective prevention activities, exploring the building of social capital and connectedness in recently arrived communities. While not specifically addressing mental disorders, this funding was based on the concept of mental wellbeing, and is essentially indistinguishable methodologically, from the over $30 million annual investment in settlement support programs funded by DIAC, for which evaluation with respect to mental health is lacking.

Overall, with respect to In the funded research identified there was a strong focus on recently arrived groups, especially refugees. There is little continuing work on the longer-term resident immigrant communities, including follow-up work from studies that have demonstrated higher rates of suicide in certain immigrant groups [44-46,48,62].

#### Current Research Activities

The final component in assessing Australian research was a survey of university departments and research groups. The response rate was 33%. From 277 surveyed institutions, only nine relevant projects were identified. Again there was a continuation of work on existing themes of peri-natal depression and asylum seekers. A few studies on health promotion and mental illness literacy and pathways to care could potentially contribute much needed information.

#### Research Activities Summary

Collectively, the body of research published and the work currently conducted is very limited in scale and scope. Little is known about the prevalence of depression, risk factors and protective factors, cultural concepts of depression and attitudes to depression, pathways to care, and uptake and effectiveness of existing interventions in relation to ethnic minority communities. For depression in ethnic minority communities there is effectively no evidence-base to support mental health policy development and service design, and there is virtually no evidence concerning effectiveness of services currently provided or regarding particular treatment approaches and models of service.

### Service Provision

The response rate to the services survey was 28%. Overall, 101 programs were reported to us as being relevant to ethnic minority communities (from 97 organisations out of the 422 that responded to the survey). Among these programs, the focus on depression or mental health and ethnic minority communities was variable. We classified programs according to their relevance in addressing depression in ethnic minority communities (Table 3). Six programs directly addressed this issue while 40 were more broadly focused on mental health in ethnic minority communities. Only three mainstream mental health programs and two depression-focused programs reported cultural adaptations to respond to the particular needs of ethnic minority groups. Most organisations targeted multiple communities with the same program with little evidence of adaptation. The most frequently targeted communities were Chinese, Vietnamese, Spanish-speaking, Italian and Greek – all of which represent large and longer-term resident ethnic minority communities in Australia. This service focus contrasts sharply with the research focus, which is very much on recently arrived groups.

**Table 3:** Program types and number of programs described

**Program Types**		**Number**
**Type A. **	Ethnic minority group programs that address depression specifically	6
**Type B. **	Ethnic minority group programs that address mental health issues with no specific focus on depression	40
**Type C. **	Ethnic minority group programs which addressed community development issues but not directly mental health problems and depression	13
**Type D. **	Mainstream depression program with adaptation for ethnic minority communities	5
**Type E. **	Mainstream depression program stating ethnic communities are not excluded but there was little to suggest program adaptation for including ethnic communities	2
**Type F. **	Mainstream mental health programs where there is a specific adaptation for including ethnic communities	3
**Type G. **	Mainstream mental health programs, stating ethnic communities are not excluded but there was little to suggest program adaptation for including ethnic communities	22
**Type H. **	Mainstream community development stating ethnic communities are not excluded but there was little to suggest program adaptation for including ethnic communities	10

We reduced the wide range of information provided for the 101 programs that were reported to us by thematic/content analysis. Programs were analysed in terms of barriers, strategies and required supports, role in addressing depression in ethnic minority communities, and involvement of ethnic minority consumers and caregivers and other ethnic minority community members. Five key themes – *Information Needs, Need for Organisation and Integration, Resource Needs, Process Barriers*, and *Program Focus *– were identified through progressive analytical coding refinements carried out by one researcher in regular consultation and review with the other researchers.

Various *Information Needs *were reported by services, including the need for research that could inform their work. The most frequent aspect of information needs was the need for *local *data, both on communities and on resources related to mental health, including information regarding demography, social conditions, epidemiology of depression, mental health issues within ethnic minority communities, the availability of services and supports, 'best practice' in service delivery to ethnic minority communities, 'working' models that involve ethnic minority communities, available ethnic and bilingual service providers, and cultural issues and community understandings of depression.

*Need for Organisation and Integration *involved the establishment and maintenance of collaborative links, e.g., between mainstream health and mental health services and ethno-specific community organisations. This included ethno-specific organisations providing assistance in the development of culturally appropriate programs, and mainstream organisations assisting ethnic community organisations with acquiring funds for their activities and promoting mental health activities initiated by ethno-specific organisations. Ethno-specific organisations saw a role in advocating on behalf of ethnic minority communities in relation to mental health services, program development and implementation. Despite the expressed need for collaborative work, ethno-specific services felt that they were not recognised ass having a 'legitimate place' within the health care system. On the other hand, mental health services reported a lack of coordination in their attempts to reach ethnic minority communities with their programs. There was the stated need for some 'external process' to organise and support partnership processes, particularly between ethno-specific organisations and mainstream mental health services.

General Practice was seen as not sufficiently involved with the broader community-based initiatives to deal with mental health issues and there was a stated need for general practitioners to be more effectively linked to broader community strategies for mental health. Bilingual medical professionals were considered important for this (together with bilingual allied mental health workers and specifically psychologists).

There was also an expressed need for greater involvement of consumers, caregivers and other community members from ethnic backgrounds to provide input into programs. Current input, where it occurred, was predominantly through informal consultation.

*Resource Needs *included calls for increased general funding and project-specific funding to support activities within programs, and for the development and dissemination of translated materials in languages other than English. Several respondents considered linkages with State transcultural mental health centres to be useful in supporting their programs and for accessing multilingual mental health information. Several respondents expressed a need for bilingual workers and workers within the mental health system with cultural awareness or knowledge of particular ethnic minority communities. Lack of culturally appropriate services for referral or collaborative casework was considered to be a problem by several community-based organisations.

The main *Process Barriers *identified by ethno-specific services included language barriers; high caseloads preventing workers from participating in community development; lack of bilingual staff; threats to program continuity; and low literacy levels in some communities affecting program reach. Limited program reach within particular communities and subgroups within a community (e.g. men) was emphasised by mainstream mental health programs. Lack of program funding was a common concern. Barriers within mainstream organisations (as described by ethno-specific organisations) included attitudinal issues, e.g., mainstream health service staff were considered 'uncooperative' and mental health workers were described by some as lacking specific commitment to the issues of mental health of ethnic minority communities. Some respondents suggested an 'action plan' to reduce racism within the hospital and community health system. Lack of long-term funding was thought to lead to short-term planning and incapacity for longer-term activities, which were perceived as more effective for ethnic minority communities. In relation to research barriers, community-based programs emphasised difficulties in working collaboratively with university-based researchers due to conflict over project ownership and intellectual property. Further, funding bodies were perceived as not having a commitment to prioritising the issues of mental health in ethnic minority communities.

Mainstream organisations expressed the view that ethnic minority communities 'did not trust' them and they perceived this as presenting a 'cultural barrier' to providing services. It was considered that some communities 'had different understandings of the service delivery system' and 'a lack of awareness regarding treatments', such as counselling and psychotherapy, which diminished access to services. Several responses indicated the need to develop promotional materials in languages other than English, to participate in ethnic community events and to encourage ethnic minority community volunteer participation in their service programs.

*Program Focus *relates to activities and roles, either carried out or intended. There was a call to address issues of 'racism, stereotyping and discrimination' through community education or media campaigns. Mass media, ethnic media and direct information and education sessions were the main suggested forms for information provision outside direct contact through casework. This activity was seen as a means of preventing the circumstances that may contribute to depression in members of ethnic minority communities. Within ethnic minority communities, community education was seen as a key strategy for a number of outcomes, including reducing stigma associated with depression (and other mental illness), increasing knowledge and recognition of depression, and increasing community understanding of relevant health services and other available support generally, thereby improving access to care. Further, education addressing the lack of English proficiency was seen as an important strategy for improving access to a variety of supports, services, employment and information.

Education was also seen to be important in the mainstream health system, for primary care workers as well as for mental health professionals. In the view of respondents, such education should provide training on cross-cultural or cultural issues in general. Ethno-specific organisations and ethnic primary care workers were seen to require training on mental health issues in order to facilitate more effective working arrangements in relation to mental health problems. Another form of training was to 'skill' caregivers from ethnic minority communities in developing supports for their compatriot peers. At the same time, caregivers were considered as a group 'at risk' due to their highly demanding role, and in need of additional attention by services. Responses suggested the need to develop ethno-specific carer support groups, and to provide information, material assistance, respite services, counselling and referral services.

General practice was also seen as a target for training on cultural issues, although the one program attempting this indicated disappointing attendance by general practitioners.

Supporting the 'settlement' of the recently arrived was seen as an important strategy to prevent depression. There was also a repeated suggestion to link members of ethnic minority communities to social support activities, community cultural events and recreation programs. Programs developed for elderly persons from ethnic minority communities were aimed at relieving their isolation and at providing direct assistance such as referral to aged care and social services.

Direct clinical services, such as counselling, psychotherapy, psychiatric case management, psychological rehabilitation, day activity programs, self-help and mutual support groups for those with mental disorders, were all reported by mainstream mental health organisations. These were regarded as available to the whole of the community with no particular adaptations of programs to accommodate the varied needs of ethnic minority communities. Following 'best practice' was seen as a means of improving the quality of such services to members of ethnic minority communities, although there was a stated need to identify what constitutes 'best practice' in this context.

Beyond direct and supportive services and education activities, many organisations, particularly ethnic minority community services, were perceived as having a role in advocating on behalf of the community, in contributing to the planning, development and implementation of programs and services, and in working towards the development and implementation of policy.

## Discussion

The analysis of policies revealed that, collectively, they provide comprehensive direction for service reform and practice in relation to ethnic minority communities. Australian States with longer-term commitment to addressing the problems of ethnic minority groups have specialised transcultural mental health policies and a range of strategies in place. Policy and policy implementation should be judged in the context of the broader reforms undertaken in the Australian mental health system [63]. There have been substantial competing priorities in the context of broadly under-funded mental health services [64], particularly as they have moved from hospital-based to community-based services. Moreover, the emphasis initially under the National Mental Health Strategy was strongly interpreted as addressing 'severe' mental disorders to the near exclusion of the common mental disorders [65]. Along with these changes there has been a shift in the structure and skills in the mental health workforce. The level of attention to population diversity issues is better appreciated within the broader context of the very substantial systemic reforms that have occurred in mental health services in the past decade.

Nevertheless there is clearly a need to integrate the disparate policy statements from Commonwealth and State and Territories governments into a more coherent strategy. It is our view that policy documents should be framed with diversity considered as a core characteristic of the Australian community, rather than a special issue requiring later ('afterthought') adjustments of mainstream models of care. A key barrier to policy reforms is that information on mental health, service delivery, and effectiveness of services in relation to Australia's ethnic minority communities is lacking. The promotion of research is of course essential in formulating evidence-based policies, strategies and programs.

The scale and diversity of research on ethnic minority communities and depression in Australia is extremely limited. Research is needed in order to develop a systemic approach to depression in ethnic minority communities and to create an evidence-base for the design and targeting of interventions in accordance with Commonwealth and State policy intentions. The low level of research on ethnic minority communities is surprising given the multicultural composition of the Australian community. The currently available research provides limited information on some ethnic minority communities and often this information is not relevant to the development of mental health strategies. The information needed includes population prevalence of common mental disorders, knowledge of common risk and protective factors affecting incidence and chronicity of disorders, mental health literacy and pathways to care, the community-general practice interface, effectiveness of mental health promotion approaches, and evaluation of integrated mental health care models that include both public and private specialist mental health services and the primary care system.

In addition, the multicultural composition of the community affords opportunities for theory-driven research into the interaction between culture and psychopathology, migration and psychopathology, and cultural factors affecting treatment efficacy and effectiveness (including pharmacological approaches).

There is a significant gap between the information needs as expressed by service providers and the scope and focus of the research that is being done. Failure to carry out the research that is needed perpetuates population health inequalities and inequities in mental health service provision.

With respect to services and programs five key themes were identified in the responses to the survey. These were *Information Needs, Need for Organisation and Integration, Resource Needs, Process Barriers*, and *Program Focus*. These are summarised above and need not be repeated here. Addressing the variety of issues covered under these themes requires approaches that constructively address the issues and particular agendas of a number of key players: ethno-specific welfare and social services organisations, policy makers and program funding bodies, primary care and general practice, specialist mental health services, consumer, carer and community organisations, research groups and education and training organisations. There is no simple way to integrate such a range of agendas. Such integrated approaches are being promoted and pursued by state transcultural mental health centres and by the national network Multicultural Mental Health Australia.

## Conclusion

In Australia considerable policy attention has been devoted to the problem of mental illness in, and provision of services to, a multicultural population. Several key problems remain. The first is that the fundamental reality of the cultural and linguistic diversity of the population has not found its way into the consciousness of policy-makers, so that the problems of ethnic minority communities (and other population sub-groups) are generally tacked on in 'special needs' sections of the policy in question. Suggested solutions are then inevitably add-ons after key policy decisions are made. The issue of population diversity is simply not a significant consideration in the framing of policy directions. The second is that policy implementation lags far behind policy intentions. Much of what policy documents state about necessary changes in the structure and operations of mental health services in relation to ethnic minority communities has not, and will not, see the light of day. The issue of accountability for policy implementation requires considerable attention in this as in many other areas. The third, and in many ways the most fundamental, problem is the lack of systematic rigorous evidence for policy and practice. The research base is extremely limited. Innovative services approaches are almost never evaluated. From the perspective of service development and reform one of the more wasteful and negligent practices is the failure by governments to scale up innovative services that have been demonstrated to be effective.

## Competing interests

The author(s) declare that they have no competing interests.

## Authors' contributions

IHM contributed to the conception and design of the study, securing research funding, interpretation of results and writing. SK contributed to the design of the study, securing research funding, interpretation of results and writing. RK managed the process of data collection and analysis, with input throughout from IHM and SK, and wrote early drafts of study results. Minas completed the final version of the paper.

## References

[B1] Ustun TB, Ayuso-Mateos JL, Chatterji S, Mathers CD, Murray CJL (2004). Global burden of depressive disorders in the year 2000.. British Journal of Psychiatry.

[B2] Murray CJL, Lopez AD (1996). The Global Burden of Disease: a Comprehensive Assessment of Mortality and disability from diseases, injuries and risk factors in 1990 and projected to 2020.

[B3] Mathers CD, Vos ET, Stevenson CE, Begg J (2000). The Australian burden of disease study: measuring the loss of health from diseases, injuries and risk factors. Medical Journal of Australia.

[B4] Mental Health and Special Programs Branch (Commonwealth) (2000). National Action Plan for Promotion, Prevention and Early Intervention for Mental Health 2000.

[B5] Mental Health and Special Programs Branch (Commonwealth) (2000). Promotion, Prevention and Early Intervention for Mental Health 2000 - A Monograph.

[B6] Depression Drafting Group (Commonwealth) (2000). National Action Plan for Depression.

[B7] Commonwealth Department of Health and Aged Care (2000). Life: a framework for prevention of suicide and self-harm in  Australia.

[B8] Beyondblue - The National Depression Initiative http://www.beyondblue.org.au.

[B9] Mental Health Branch (Commonwealth) (1996). National Standards for Mental Health Services.

[B10] Multicultural Mental Health Australia http://www.mmha.org.au.

[B11] Mitchell P, Malak A, Small D (1998). Bilingual professionals in community mental health services.. Aust N Z J Psychiatry.

[B12] Ziguras S, Klimidis S, Lewis J, Stuart G (2003). Ethnic matching of clients and clinicians and use of mental health services by ethnic minority clients. Psychiatric Services.

[B13] Marsella AJ, Sartorius N, Jablensky A, Fenton FR, Kleinman A, Good B (1985). Cross-cultural studies of depressive disorders: an overview. Culture and Depression: Studies in the Anthropology and Cross-Cultural Psychiatry of Affect and Disorder.

[B14] Kirmayer LJ (2001). Cultural variations in the clinical presentation of depression and anxiety: implications for diagnosis and treatment. J Clin Psychiatry.

[B15] Draguns JG, Triandis HC, Draguns JG (1980). Disorders of clinical severity. Handbook of Cross-Cultural Psychology Psychopathology.

[B16] Yap PM (1951). Mental diseases peculiar to certain cultures: a survey of comparative psychiatry. Journal of Mental Science.

[B17] Westermeyer J, Janca A (1997). Language, culture and psychopathology: conceptual and methodological issues. Transcultural Psychiatry.

[B18] Westermeyer J (1985). Psychiatric Diagnosis across cultural boundaries. American Journal of Psychiatry.

[B19] Kleinman A (1977). Depression, somatization and the new cross-cultural psychiatry. Social Science and Medicine.

[B20] Parker G, Gladstone G, K.T. C (2001). Depression in the planet's largest ethnic group: the Chinese. The American Journal of Psychiatry.

[B21] Altarriba J, Santiago-Riviera (1994). Current perspectives on using linguistic and cultural factors in counselling the Hispanic client. Professional Psychology: Research and Practice.

[B22] Del Castillo LR (1970). Effects of interpreters on the evaluation of psychopathology in non-English speaking patients. American Journal of Psychiatry.

[B23] Marcos LR, Alpert M, Urcuyo L (1973). The effect of interview language on the evaluation of psychopathology in Spanish-American schizophrenic patients. American Journal of Psychiatry.

[B24] Marcos LR (1979). Effects of interpreters on the evaluation of psychopatology in non-English speaking patients. American journal of Psychiatry.

[B25] The Social Science and Data Archives (SSDA). The Social Science and Data Archives (SSDA). http://assda.anu.edu.au/.

[B26] Mental Health Division Department of Health Government of Western Australia (2001). A Transculturally Orientated Mental Health Service for Western Australia.

[B27] Mental Health Branch (Queensland) (1995). Non-English Speaking Background Mental Health Policy Statement.

[B28] NSW Health Department (1999). Caring for Mental Health in a Multicultural Society - A Strategy for the Mental Health Care of People from Culturally and Linguistically Diverse Backgrounds.

[B29] Psychiatric Services Branch (Victoria) (1996). Improving Services for People from a Non-English Speaking Background.

[B30] Ziguras SJ (1997). Implementation of ethnic health policy in community mental health centres in Melbourne. Aust N Z J Public Health.

[B31] Psychiatric Services Division (Victoria) (1996). Victoria's Mental Health Service: The Framework for Service Delivery: Better Outcomes through Area Mental Health Services.

[B32] Steel Z, Silove D, Bird K, McGorry P, Mohan P (1999). Pathways from war trauma to posttraumatic stress symptoms among Tamil asylum seekers, refugees, and immigrants. Journal of Traumatic Stress.

[B33] Steel Z, Silove DM (2001). The mental health implications of detaining asylum seekers. Med J Aust.

[B34] Steel Z, Silove D, Phan T, Bauman A (2002). Long-term effect of psychological trauma on the mental health of Vietnamese refugees resettled in Australia: a population-based study. Lancet.

[B35] Sultan A, O'Sullivan K (2001). Psychological disturbances in asylum seekers held in long term detention: a participant-observer account. Medical Journal of Australia.

[B36] Silove D, Steel Z, McGorry P, Mohan P (1998). Trauma exposure, postmigration stressors, and symptoms of anxiety, depression and post-traumatic stress in Tamil asylum-seekers: comparison with refugees and immigrants. Acta Psychiatr Scand.

[B37] Silove D, Sinnerbrink I, Field A, Manicavasagar V, Steel Z (1997). Anxiety, depression and PTSD in asylum-seekers: assocations with pre-migration trauma and post-migration stressors. British Journal of Psychiatry.

[B38] Matthey S, Barnett BE, Elliott A (1997). Vietnamese and Arabic women's responses to the Diagnostic Interview Schedule (depression) and self-report questionnaires: cause for concern. Aust N Z J Psychiatry.

[B39] Matthey S, Silove D, Barnett B, Fitzgerald MH, Mitchell P (1999). Correlates of Depression and PTSD in Cambodian women with young children: a pilot study. Stress Medicine.

[B40] Stuchbery M, Matthey S, Barnett B (1998). Postnatal depression and social supports in Vietnamese, Arabic and Anglo-Celtic mothers. Social Psychiatry and Psychiatr Epidemiology.

[B41] Barclay L, Kent D (1998). Recent immigration and the misery of motherhood: a discussion of pertinent issues. Midwifery.

[B42] Nahas V, Amasheh N (1999). Culture care meanings and experiences of postpartum depression among Jordanian Australian women: a transcultural study. Journal of Transcultural Nursing.

[B43] Barnett BE, Matthey S, Boyce P (1999). Migration and motherhood: a response to Barclay and Kent (1998). Midwifery.

[B44] Taylor R, Morrell S, Slaytor E, Ford P (1998). Suicide in urban New South Wales, Australia 1985-1994: Socio-economic and migrant interactions. Soc Sci Med.

[B45] Burvill PW (1998). Migrant suicide rates in Australia and in country of birth. Psychological Medicine.

[B46] Burvill P (1995). Suicide in the multiethnic elderly population of Australia, 1979-1990. International Psychogeriatrics.

[B47] Domino G, Niles S, Raj SD (1993). Attitudes towards suicide: a cross-cultural comparison of Singaporean and Australian university students. Omega.

[B48] Morrell S, Taylor R, Slaytor E, Ford P (1999). Urban and rural suicide differentials in migrants and the Australian-born, New South Wales, Australia 1985-1994. Social Science and Medicine.

[B49] Oei PS, Notowidjojo F (1990). Depression and loneliness in overseas students. The International Journal of Social Psychiatry.

[B50] Williams H, Carmichel A (1991). Depression in mothers and behaviour problems with their preschool children. J Paediatr Child Health.

[B51] Klimidis S, Stuart G, Minas IH, Ata AW (1994). Immigrant status and gender effects on psychopathology and self-concept measures in adolescents: A test of the migration-morbidity hypothesis. Comprehensive Psychiatry.

[B52] Mildred H, Paxton SJ, Wertheim EH (1995). Risk factors for eating disorders in Greek- and Anglo-Australian adolescent girls. International Journal of Eating Disorders.

[B53] McLennan W (1998). Mental Health and Wellbeing: Profile of Adults, Australia 1997.

[B54] Comino EJ, Silove D, Manicavasagar V, Harris E, Harris MF (2001). Agreement on symptoms of anxiety and depression between patients and GPs: the influence of ethnicity. Family Practice.

[B55] Knox SA, Brit H (2002). A comparison of general practice encounters with patients from English-speaking and non-English speaking backgrounds. Medical Journal of Australia.

[B56] Tse T (2002). Islamic community worker program for the management of depression. Australian e-Journal for the Advancement of Mental Health.

[B57] Alarcon H (2001). Psychological analysis of unemployment, self-esteem and depression among professional migrants.. Faculty of Education.

[B58] Croft S (1990). Depression in Vietnamese adolescents.. Dept not indicated.

[B59] Kapelus S (1997). Levels of depression and anxiety in a sample of South African migrants: a measure of adaptability.. Psychology Department.

[B60] Small R (2000). Mothers in a new country (MINC): an Australian study of Vietnamese, Turkish, Filipino women's experience of maternity care and of maternal depression after childbirth.. Centre for the Study of Mothers' and Children's Health.

[B61] von Treuer K (1999). Prevalence, social and biological risk factors between Australian-born Australian women and Vietnamese-born Australian women with post-natal depression.. Department of Psychology.

[B62] McDonald B, Steel Z (1997). Immigrants and Mental Health: An Epidemiological Analysis.

[B63] Commonwealth Department of Health and Aged Care (2000). National Mental Health Report 2000: Sixth Annual Report. Changes in Australia's Mental Health Services under the First National Mental Health Plan of the National Mental Health Strategy..

[B64] Andrews G, Hall W, Teesson M, Henderson S (1999). The Mental Health of Australians.

[B65] Betts V, Thornicroft G (2001). International Mid-term Review of the Second National Mental Health Plan for Australia..

[B66] Australian Health Ministers (1992). National Mental Health Policy.

[B67] Australian Health Ministers (1991). Mental Health Statement of Rights and Responsibilities..

[B68] Australian Health Ministers (1998). Second National Mental Health Plan.

[B69] Mental Health Branch (Commonwealth) (2000). Mental Health Promotion and Prevention National Action Plan under the Second National Mental Health Plan: 1999-2003.

[B70] Mental Health Branch (Commonwealth) (1997). Youth Suicide in  Australia: the National Youth Suicide Prevention Strategy.

[B71] ACT Department of Health and Community Care (1998). The Future of Mental Health Services in the Australian Capital Territory - Moving Towards 2000 and Beyond - A Whole of Territory Strategic Plan 1998-2001.

[B72] NSW Health Department (1998). Caring for Mental Health -  A Framework for Mental Health Care in NSW.

[B73] NSW Health Department (1998). Caring for Older People's Mental Health - A Strategy for the Delivery of Mental Health Care for Older People in NSW.

[B74] NSW Health Department (2001). Getting in Early - A Framework for Early Intervention and Prevention in Mental Health for Young People in NSW.

[B75] Tasmania Department of Health and Human Services (2001). Tasmanian Multicultural Policy.

[B76] Tasmania Department of Health and Human Services (2001). Rural Mental Health Plan 2001-2004.

[B77] Tasmania Mental Health Services (1999). A Plan for Now and the Future - Strategic Plan for 1999-2002..

[B78] Smith G, McCavanagh D, Williams T, Lipscombe P (1996). Making a Commitment: The Mental Health Plan for Western Australia.

[B79] Queensland Health (1996). Ten Year Mental Health Strategy for Queensland.

[B80] Queensland Health (2000). Queensland Health Multicultural Policy Statement.

[B81] Mental Health Services (South Australia) (2000). A New Millenium - A New Beginning - Mental Health in South Australia.

[B82] Psychiatric Services Branch (Victoria) (2002). New Directions for Victoria's Mental Health Services: the Next Five Years.

